# Electrospinning and Partial Etching Behaviors of Core–Shell Nanofibers Directly Electrospun on Mesh Substrates for Application in a Cover-Free Compact Air Filter

**DOI:** 10.3390/nano14131152

**Published:** 2024-07-05

**Authors:** Yujung Lee, Seungwoo Jung, Ji Sun Yun

**Affiliations:** New Growth Materials Division, Korea Institute of Ceramic Engineering and Technology, 101 Soho-ro, Jinju 52851, Republic of Korea; ujung0319@kicet.re.kr (Y.L.); swjung@kicet.re.kr (S.J.)

**Keywords:** electrospinning, core–shell-structured nanofiber, mesh substrate, air filter, propylene glycol monomethyl ether acetate

## Abstract

The exposure of workers to propylene glycol monomethyl ether acetate (PGMEA) in manufacturing environments can result in potential health risks. Therefore, systems for PGMEA removal are required for indoor air quality control. In this study, core–shell zeolite socony mobil-5 (ZSM-5)/polyvinylpyrrolidone–polyvinylidene fluoride nanofibers were directly electrospun and partially wet-etched on a mesh substrate to develop a cover-free compact PGMEA air filter. The electrospinning behaviors of the core–shell nanofibers were investigated to optimize the electrospinning time and humidity and to enable the manufacture of thin and light air-filter layers. The partial wet etching of the nanofibers was undertaken using different etching solvents and times to ensure the exposure of the active sites of ZSM-5. The performances of the ZSM-5/PVDF nanofiber air filters were assessed by measuring five consecutive PGMEA adsorption–desorption cycles at different desorption temperatures. The synthesized material remained stable upon repeated adsorption–desorption cycles and could be regenerated at a low desorption temperature (80 °C), demonstrating a consistent adsorption performance upon prolonged adsorption–desorption cycling and low energy consumption during regeneration. The results of this study provide new insights into the design of industrial air filters using functional ceramic/polymer nanofibers and the application of these filters.

## 1. Introduction

Recently, because of the high awareness of indoor and outdoor air quality, several studies have attempted to overcome the environmental and health problems caused by indoor air pollution [1,2]. Indoor pollutants primarily comprise particulate matter, nitrogen dioxide, volatile organic compounds (VOCs), and sulfur dioxide, and much research is focused on the removal of pollutants from contaminated air [3]. The removal of VOCs, the most common air pollutants, which lead to various harmful diseases, even at low concentrations, is a challenging process. Although VOCs that adversely affect human health are emitted from several artificial sources, most studies on VOCs focus on biogenic aromatic species such as benzene, toluene, ethylbenzene, and xylene (BTEX), which are common indoor pollutants [4]. Significant amounts of highly toxic aromatic VOCs, for example, BTEX, are generated from building materials, household products, food processing, preservation processes, chemical substances used in chemical factories, and paint manufacturing plants [5,6,7]. The chemical solvent propylene glycol monomethyl ether acetate (PGMEA) is widely used for manufacturing semiconductor displays; it improves the performance and reliability of the final product by eliminating impurities during manufacturing [8,9]. Although PGMEA is less toxic than BTEX compounds, the exposure of workers to PGMEA in manufacturing environments can result in potential health risks, including respiratory and eye irritation and kidney dysfunction [10,11]. Furthermore, recovered and recycled PGMEA is a higher-value resource than BTEX compounds. Therefore, specialized technology for PGMEA removal should be developed for indoor air-quality control.

Numerous studies have investigated elimination strategies for VOCs for indoor air-quality control, for instance, the use of photocatalytic technologies and air-filter materials such as antimicrobial materials, nanofiber filters, and systems based on metal–organic frameworks [12,13,14]. However, these materials and approaches have mainly been used to maximize the atmospheric removal of BTEX compounds by adsorption. Moreover, although multilayer filtration enhances the adsorption performance of filters, such systems are associated with several disadvantages such as considerably lower air permeabilities, higher overall filter volumes, and extended processing times and costs when compared with single-layer filter systems [15,16]. The literature on atmospheric filters for VOCs indicates that new and innovative air filters that selectively eliminate and recover PGMEA for industrial air-filter applications should be urgently established. Efficient PGMEA air-filter materials that are light and thin and that permit facile functional group incorporation are expected to have a wide range of applications. Thus, the design and development of these materials are challenging yet vital aspects of air-filter research.

In this study, cover-free compact PGMEA air-filter materials containing core–shell-structured zeolite socony mobil-5 (ZSM-5)/polyvinylpyrrolidone (PVP)–polyvinylidene fluoride (PVDF) nanofibers on a mesh substrate were constructed by direct electrospinning and partial wet etching. Three primary aspects were considered during the design of the materials and manufacturing processes: (1) the adsorbate–adsorbent interaction mechanism—given the excellent selective adsorption of PGMEA by zeolite particles, the porous material ZSM-5, which is composed of a 10-ring cage structure (dimensions 5.1 Å × 5.5 Å) and has high acidity (SiO_2_/Al_2_O_3_ = 23), was chosen for the selective adsorption of PGMEA molecules, which exhibit sizes of 4.0–5.0 Å and have low polarities [17]; (2) the characteristics of the PGMEA air-filter material—as electrospinning and partial wet etching were to be employed to fabricate the base filter layer and expose the active sites of the core–shell zeolite/PVP-PVDF nanofibers [18], the PGMEA air-filter material manufactured in this study needed to be flexible, light, and thin and allow effortless functional group introduction; and (3) the properties of the cover-free compact air filter, which was synthesized by directly electrospinning nanofibers on a mesh substrate that functioned as both a protective and support layer. Based on these three main design strategies, the core–shell ZSM-5/PVP-PVDF nanofibers, which can be electrospun in a single step according to the surface energy difference between PVP and PVDF [19], were directly electrospun on the mesh substrate and then partially wet-etched. This study suggests the possibility of developing a high-performance air filter that not only does not require an additional protective layer but is also a flexible, lightweight, thin, and compact structure. Furthermore, key factors impacting the PGMEA removal by the air filter were analyzed to optimize the synthesis of these nanofibers, and guidelines were devised to optimize PGMEA-selective adsorption process conditions.

## 2. Materials and Methods

### 2.1. General Experimental Details

*N*,*N*-Dimethylformamide (DMF, 99.8%, Sigma-Aldrich, St. Louis, MO, USA), acetone (99.9%, Sigma-Aldrich, St. Louis, MO, USA), ZSM-5 (CBV 2314, SiO_2_/Al_2_O_3_ = 23, Zeolyst International, Conshohocken, PA, USA), PVP (molecular weight = 1,300,000, Alfa Aesar, Haverhill, Ma, USA), and PVDF (Alfa Aesar, Haverhill, Ma, USA) were purchased.

The morphologies of the core–shell ZSM-5/PVP-PVDF and ZSM-5/PVDF nanofibers were characterized using field-emission scanning electron microscopy (SEM) (FE-SEM, MIRA3 LM, TESCAN, Brno, Czech Republic). Optical images of the ZSM-5/PVP-PVDF nanofibers were recorded with an optical microscope (BX53MRF-S, OLYMPUS, Tokyo, Japan). The basis weight of the ZSM-5/PVDF nanofibers was the weight of the nanofibers alone, except for a substrate weight per unit area of the air filters. The specific surface areas of the ZSM-5/PVDF nanofibers were analyzed with an ASAP 2000 instrument (Micromeritics Inc., Norcross, GA, USA) and calculated using the Langmuir and Freundlich methods based on N_2_ adsorption at liquid N_2_ temperature. The air permeabilities of the ZSM-5/PVDF nanofibers were measured with an air-permeability tester (FX3300, TEXTEST Inc., Columbus, GA, USA). Gas chromatography (GC) was conducted with a GC-2010 Plus (SHIMADZU, Kyoto, Japan).

### 2.2. Preparation of ZSM-5/PVP-PVDF Precursor Solution

Core–shell ZSM-5/PVP-PVDF nanofibers were manufactured by using a reported method [18] with a precursor solution in a mixed solvent consisting of DMF and acetone (1:1 wt/wt; where DMF improved the electrospinnability of the solution and acetone enhanced the solubilities of the polymers). A ZSM-5/PVP precursor solution was prepared by sequential addition of ZSM-5 (2 g) and PVP (1 g) to a mixture of DMF and acetone (1:1 wt/wt, 8 g). ZSM-5/PVDF precursor solution was prepared by the sequential addition of ZSM-5 (1 g) and PVDF (1 g) to a mixture of DMF and acetone (1:1 wt/wt, 8 g). Both precursor solutions were sonicated for 30 min in a bath and then tip-sonicated for 10 min for 15 cycles, followed by vigorous stirring for 24 h. The ZSM-5/PVP and ZSM-5/PVDF precursor solutions were then combined and mixed vigorously for 6 h to obtain the ZSM-5/PVP-PVDF precursor solution.

### 2.3. Generation of ZSM-5/PVDF Nanofibers by Electrospinning

**Figure 1:**
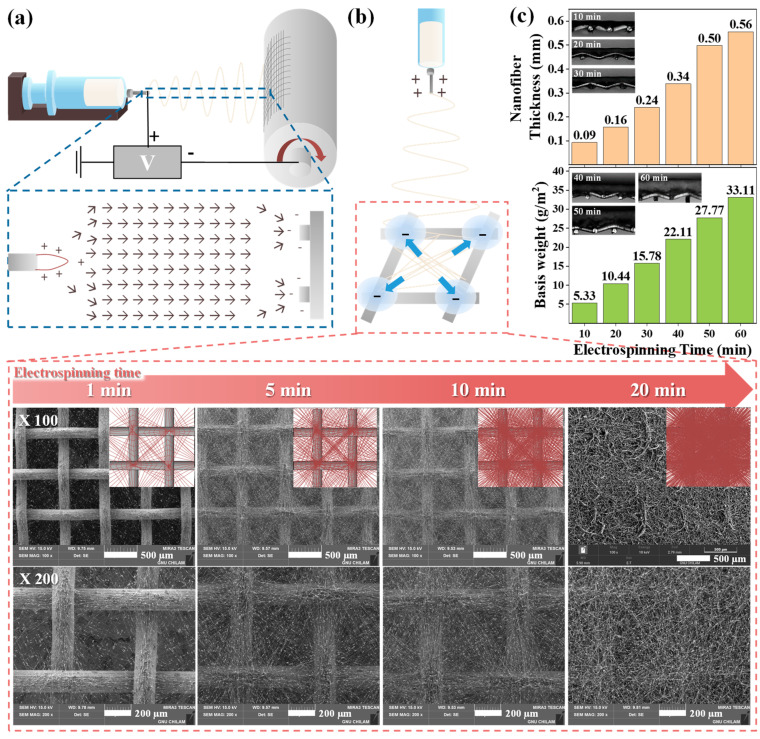
(**a**) Schematic illustrating the shapes of the electric field formed between the needle tip and the collector during the electrospinning of nanofibers on the mesh substrate. (**b**) Schematics and SEM images of the morphologies of the core–shell ZSM-5/PVP-PVDF nanofibers settled onto the mesh substrate at different electrospinning times. (**c**) Thicknesses, basis weights, and optical images of ZSM-5/PVP-PVDF nanofibers formed at different electrospinning times.

Homogeneous ZSM-5/PVP-PVDF precursor solution was loaded into a syringe connected to a metallic needle (20 G) and electrospun directly onto the surface of a 40-mesh metal substrate fixed to a cylindrical-drum collector rotating at 250 rpm (Figure 1a). The distance between the metallic needle tip and the collector was maintained at 15 cm, and a positive voltage of 11.7 kV was applied to the metallic needle. The precursor solution was supplied at a feeding rate of 1.5 mL/h, and a relative humidity (RH) of 30–40% was retained throughout the process. After drying the electrospun ZSM-5/PVP-PVDF nanofibers at 70 °C for 24 h, hydrophilic PVP in the shell parts of the as-spun nanofibers was partially wet-etched using aqueous ethanol (25 vol%). The wet-etched nanofibers were additionally dried at 70 °C for 24 h, and the resulting partially etched nanofibers were labeled as ZSM-5/PVDF nanofibers.

### 2.4. Characterization of the Nanofibers

ZSM-5/PVDF nanofibers manufactured as described in Section 2.3 were subjected to five consecutive PGMEA adsorption–desorption cycles in a catalytic reaction system (TENG Inc., Daejeon, Republic of Korea). ZSM-5/PVDF nanofibers (0.05 g) were placed in a Pyrex glass reactor (1 cm inner diameter) with quartz wool (0.05 g) at each end, and the tube was heated for 1 h at 150 °C under Ar. After cooling, PGMEA adsorption at 35 °C was investigated by supplying PGMEA (5000 ppm) to the system at a flow rate of 150 cm^3^/min under Ar. Outflow gas concentration/inflow gas concentration (C/C0) values at different adsorption times were determined by GC analysis; the time at C/C0 = 0.05 was considered the breakthrough time. After adsorption, desorption experiments were conducted by heating the system from 35 °C to either 150 or 80 °C at 2 °C/min and maintaining the desorption temperature for 3 h. This PGMEA adsorption–desorption cycle was performed five times in a series.

## 3. Results and Discussion

The generation and characterization of core–shell ZSM-5/PVP-PVDF nanofibers electrospun on mesh substrates are shown in Figure 1. The ZSM-5/PVP-PVDF precursor solution was directly electrospun on the mesh substrate surface, with the resulting morphology of the fibers determined by the electric field shape generated between the needle tip and mesh substrate. Figure 1a depicts the process involved in electrospinning the precursor solution and its subsequent settling on the mesh substrate. The electric field lines at the collectors composed of conductive substrates separated by a void gap differ from those at the conventional collectors due to the splitting of the conductive region [20,21]. Thus, the nanofibers near the mesh frames of the patterned metal-mesh collectors, particularly at the intersection points, are more strongly charged than nanofibers in other regions, and they are pushed by the electrostatic force from the void toward the intersection points; consequently, these fibers begin to diagonally align (Figure 1b). SEM images (Figure 1b), which illustrate the morphologies of the core–shell ZSM-5/PVP-PVDF nanofibers settled on the mesh substrate at different electrospinning times, confirm that the ZSM-5/PVP-PVDF nanofibers start to settle on the mesh frame in a diagonal shape in the void gap at an electrospinning time of 1 min. When the electrospinning time was extended to 5 or 10 min, thicker nanofiber layers were generated with similar electrospinning behaviors. Electrospinning for 10 min caused the nanofibers to settle more evenly as a single layer on the mesh surface. Upon further increasing the electrospinning time, the nanofibers settled as a dense, even layer on the mesh surface, similar to deposition on a conventional collector, irrespective of the mesh substrate frame and voids. Cross-sectional images and analysis of the thicknesses of the nanofibers that were directly electrospun on the mesh substrate for different durations were analyzed by optical microscopy. The basis weights of the nanofibers were measured by determining the weight difference of the specimen before and after electrospinning (Figure 1c). Upon increasing the electrospinning time from 10 to 60 min, the thicknesses and basis weights of the nanofibers increased significantly from 0.09 mm and 5.33 g/m^2^ to 0.56 mm and 33.11 g/m^2^, respectively. Generally, as the densities of the nanofibers increased with an increase in the electrospinning time, the thicknesses and basis weights of the nanofibers increased, whereas their air permeabilities decreased rapidly. In this study, although the nanofibers fabricated with an electrospinning time of ~10 min had a morphology that was suitable for constructing air filters, to offset density reductions owing to partial etching, the nanofibers manufactured with an electrospinning time of 20 min were used.

**Figure 2:**
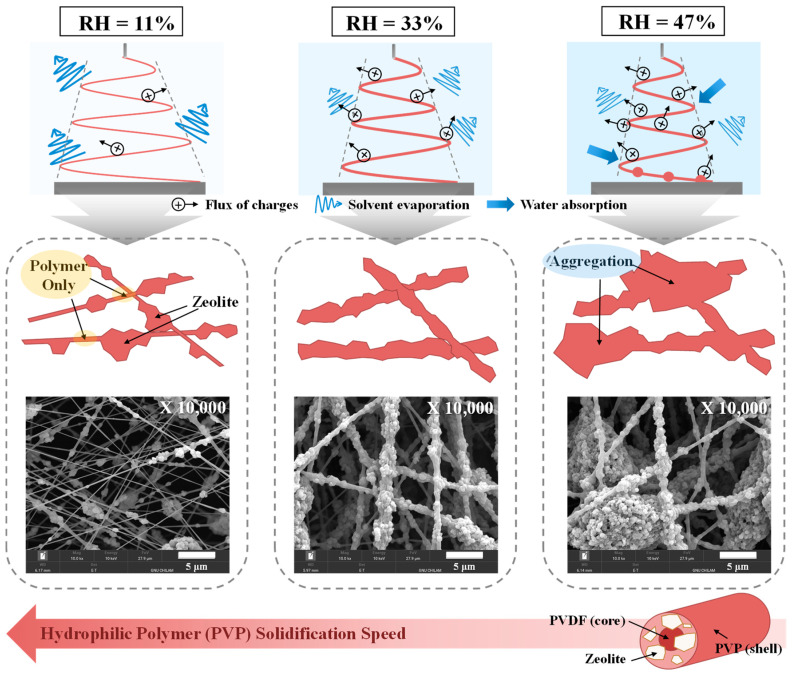
Electrospinning behaviors of ZSM-5/PVP-PVDF nanofibers manufactured at different RHs.

Given that RH substantially influences both solvent evaporation and nanofiber solidification during electrospinning [22], the behavior of the nanofibers fabricated at different RHs was studied, as shown in Figure 2. In the high electric field, water molecules in the atmosphere undergo facile ionization; thus, high-humidity conditions facilitate the discharge of electrospun jets, thereby reducing whipping movements and the solvent evaporation rate. Moreover, significant levels of water vapor absorption from the atmosphere by hydrophilic polymers, such as PVP, under high-humidity conditions enable the electrospun jets to stretch a significant distance due to the reduced nanofiber solidification rate. Notably, these phenomena have a considerable impact on the electrospinning properties of composite precursor solutions containing both ceramic and polymer species; that is, ceramic particle dispersions in electrospun nanofibers. The shells and cores of the nanofibers manufactured in this study are composed of hydrophilic (PVP) and hydrophobic (PVDF) polymers, respectively, and the hydrophilic polymers are exposed at the nanofiber surface [18]. Thus, the solidification tendencies of the nanofibers are similar to those of PVP. The SEM images presented in Figure 2 indicate that the core–shell nanofibers synthesized at a high RH of 47% contain extensively agglomerated zeolite particles. These results demonstrate that the jet readily discharges and elongates under these conditions, leading to a narrowed whipping jet motion. This, in turn, results in slower solvent evaporation and nanofiber solidification. In contrast, nanofibers manufactured at a low RH of 11% exhibit heterogeneous structures with the zeolite separated from the core–shell-structured polymer owing to rapid solvent evaporation and nanofiber solidification. Notably, the nanofibers fabricated at a RH of 33% settle as stable networks on the collector and consist of uniformly distributed zeolite particles due to optimal solvent evaporation and nanofiber solidification rates. Therefore, 30–40% RH was identified as being optimal for the electrospinning of core–shell ZSM-5/PVP-PVDF nanofibers.

**Figure 3:**
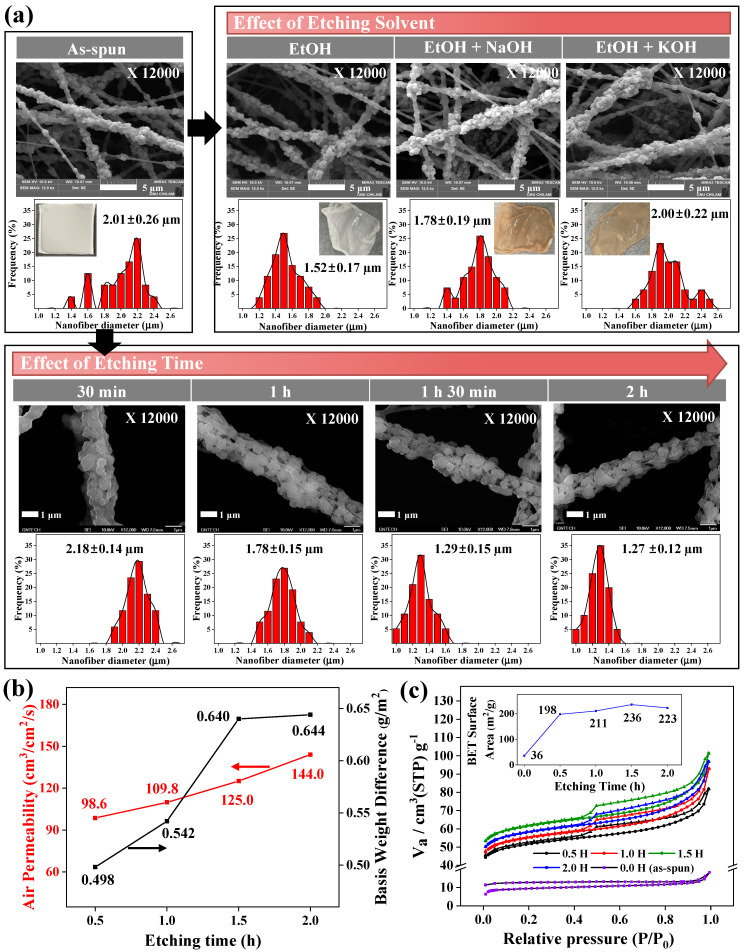
(**a**) SEM images and diameter distributions of the ZSM-5/PVDF nanofibers manufactured using different etching solvents and times. (**b**) Air permeabilities and basis weights of the ZSM-5/PVDF nanofibers synthesized using different etching times. (**c**) N_2_ adsorption–desorption isotherms and Brunauer–Emmett–Teller surface areas (inset) of the ZSM-5/PVDF nanofibers manufactured using different etching times.

The effects of etching conditions, such as the etching solvent and etching time of the PVP shell components of the nanofibers, onto the ZSM-5/PVDF nanofibers in the system are shown in Figure 3. The active sites of the ZSM-5 ceramic particles embedded in the core (PVDF)–shell (PVP) structures of the as-synthesized nanofibers were exposed by the partial wet etching of the shell components [18]. The influence of the etching solvent on the ZSM-5/PVDF nanofibers was investigated using three etching solvents: aqueous ethanol (EtOH, 25 wt%), aqueous ethanol (25 wt%) containing either NaOH (0.1 vol%; 1 M; termed EtOH + NaOH), and KOH (0.1 vol%, 1 M; termed EtOH + KOH) (Figure 3a). A previous study on HY–zeolite nanofibers indicated that of the three etching systems, the optimal etching effect was obtained with EtOH + KOH [18]. In contrast, for the ZSM-5/PVDF nanofibers, the use of EtOH containing either NaOH or KOH imparted a light reddish-brown color to the material. Additionally, the ZSM-5/PVDF nanofibers etched with EtOH containing either NaOH or KOH exhibited average diameters of 1.78 ± 0.19 and 2.00 ± 0.22 μm, respectively; thus, the diameter of the nanofibers upon etching was similar to, or significantly smaller than, those of the as-spun ZSM-5/PVDF nanofibers (average diameters of 2.01 ± 0.26 μm). In contrast, the ZSM-5/PVDF nanofibers etched with EtOH did not exhibit color changes and showcased substantially small diameters (1.52 ± 0.17 μm) and normal diameter distributions. This phenomenon can be attributed to the higher number of acid sites in the ZSM-5 (SiO_2_/Al_2_O_3_ = 23) nanofibers, synthesized herein, than in the HY–zeolite (SiO_2_/Al_2_O_3_ = 60) nanofibers used in the previous study [18]. Strong acid sites in the ZSM-5/PVDF nanofibers possibly undergo side reactions with either strong base (NaOH or KOH) in the etching solvent. Therefore, EtOH was selected as the optimal wet-etching solvent for the core–shell ZSM-5/PVP-PVDF nanofibers prepared in this study. The FT-IR spectra (Figure S1) of the ZSM-5/PVP-PVDF nanofibers before and after etching were almost similar, but only the C=O stretch peak at 1660 cm^−1^, originating from PVP, decreased after etching, showing that the PVP was well etched. When the surface morphologies and diameter distributions of the ZSM-5/PVDF nanofibers were examined at different etching times using EtOH as the wet-etching solvent (Figure 3a), an increase in the etching time from 30 min to 2 h led to a gradual decrease in the diameter of the ZSM-5/PVDF nanofibers, and the zeolite particles became more clearly observed because of increased shell–polymer etching. Although the diameters of the ZSM-5/PVDF nanofibers exhibited normal distributions regardless of the etching time, increasing the etching time to 0.5, 1, 1.5, and 2 h resulted in a decrease in the average nanofiber diameters to 2.18 ± 0.14, 1.78 ± 0.15, 1.29 ± 0.15, and 1.27 ± 0.12 µm, respectively. Additionally, due to an enhancement in the etching of the shell polymer (PVP), the air permeabilities and basis weight differences of the ZSM-5/PVDF nanofibers gradually increased from 98.6 cm^3^/cm^2^/s and 0.498 g to 144.0 cm^3^/cm^2^/s and 0.644 g, respectively, with an increase in the etching time from 0.5 to 2 h (Figure 3b). Furthermore, the ZSM-5/PVDF nanofibers, etched for 0.5, 1, 1.5, and 2 h, demonstrate specific surface areas of 198.0, 210.8, 235.9, and 222.6 m^2^/g, respectively (Figure 3c). An increase in the etching time from 0.5 to 1.5 h enhanced the etching of the PVP shell polymer, resulting in nanofibers with low diameters, high air permeabilities, large basis weight reductions, and high specific surface areas. Interestingly, the ZSM-5/PVDF nanofibers etched for 1.5 h and those etched for 2 h exhibit similar average diameters and basis weight differences; the specific surface areas of the ZSM-5/PVDF nanofibers etched for 2 h are slightly lower than those of the ZSM-5/PVDF nanofibers etched for 1.5 h. Therefore, the optimal etching time of the shell polymer PVP was identified as approximately 1.5 h. Excess etching of the PVP shell is expected to block the zeolite active sites; consequently, for maximum active site exposure, the optimal partial wet-etching time for the ZSM-5/PVDF nanofibers was determined to be 1.5 h.

**Figure 4:**
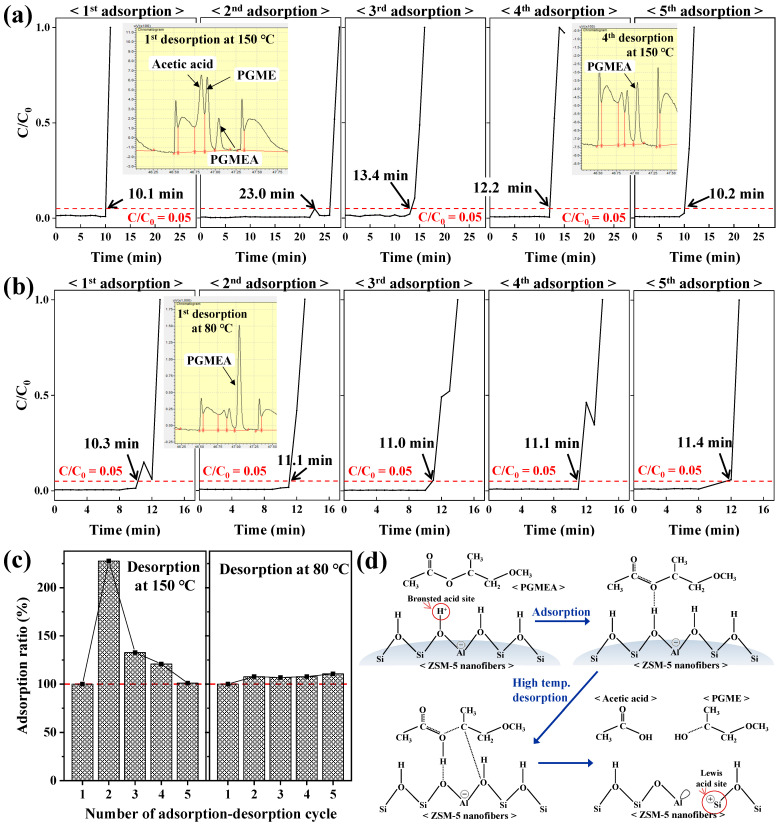
Adsorption breakthrough curves of PGMEA on ZSM-5/PVDF nanofibers for five consecutive adsorption–desorption cycles at a desorption temperature of (**a**) 150 °C and (**b**) 80 °C. (**c**) Adsorption ratio calculated from the breakthrough time (time at *C*/*C*_0_ = 0.05) during different consecutive adsorption–desorption cycles. (**d**) Schematics of the adsorption and high-temperature desorption mechanisms of PGMEA on the surface of ZSM-5/PVDF nanofibers.

The adsorption of PGMEA onto the ZSM-5/PVDF nanofiber surfaces over five consecutive adsorption–desorption cycles was examined (Figure 4). The adsorption breakthrough plots of PGMEA on the ZSM-5/PVDF nanofibers for each consecutive adsorption–desorption cycle at a desorption temperature of 150 °C are depicted in Figure 4a. The breakthrough time increases significantly from the first adsorption stage (10.1 min) to the second adsorption stage (23.0 min), decreases in the third adsorption stage (13.4 min), and continuously decreases up to the fifth adsorption stage (10.2 min), where the breakthrough time is similar to that of the first adsorption stage. In contrast, the adsorption breakthrough plots obtained at a desorption temperature of 80 °C (Figure 4b) exhibit nearly constant breakthrough times across the five cycles. Similar adsorption efficiency ratios for all the consecutive adsorption–desorption cycles at the adsorption breakthrough time (Figure 4c) confirm the abovementioned trend. The GC trace of the first desorption stage at 150 °C (Figure 4a) contains a weak PGMEA peak; in this trace, acetic acid and propylene glycol methyl ether (PGME) peaks are clearer than the PGMEA peak. In contrast, the acetic acid and PGME peaks are almost completely absent in the GC trace of the fourth desorption stage at 150 °C, whereas the PGMEA peak is clearly observed. The adsorption and subsequent high-temperature desorption of PGMEA from a surface with Brønsted acid sites can lead to the decomposition of the species into acetic acid and PGME (Figure 4d) [23].

As the active sites of the ZSM-5/PVDF nanofibers (SiO_2_/Al_2_O_3_ = 23), synthesized herein, originate from ZSM-5 particles [18], the nanofibers are composed of numerous surface Brønsted acid sites. After the first adsorption–desorption cycle at a desorption temperature of 150 °C, the Brønsted acid sites on the ZSM-5/PVDF nanofiber surface are transformed into Lewis acid sites (Figure 4d), improving the adsorption performance of the system in the second cycle. A Lewis acid site coexisting with the Brønsted acid site of the zeolite seems to accelerate the adsorption reaction due to the strong electrophilic character of the Al-COH_2_^+^ intermediate [23]. Successive adsorption–desorption cycles cause less PGMEA decomposition owing to the lack of Brønsted acid sites for conversion; consequently, the GC trace of the fourth desorption stage at 150 °C exhibits weak peaks relative to acetic acid and PGME. Furthermore, although an increase in the number of Lewis acid sites on the surfaces of the ZSM-5/PVDF nanofibers temporarily improves the adsorption efficiency of the system, the adsorption efficiency decreases gradually because of the inferior regeneration of the system during repeated adsorption–desorption cycles. In contrast, the GC trace of the fourth desorption stage at 80 °C (Figure 4b) consists of a clear PGMEA peak with very weak peaks of acetic acid and PGME, indicating that the Brønsted acid sites on the ZSM-5/PVDF nanofiber surface are appropriately retained as PGMEA does not decompose during desorption. Herein, the ZSM-5/PVDF nanofibers with surface Brønsted acid sites maintained constant adsorption efficiencies during repeated adsorption–desorption cycling, demonstrating excellent regeneration performance. Therefore, ZSM-5/PVDF nanofibers with multiple Lewis acid sites should be used in applications that require high one-time adsorption capacities, whereas ZSM-5/PVDF nanofibers with multiple Brønsted acid sites should be employed in applications requiring adsorption–desorption cycling. Moreover, the use of a low-temperature (80 °C) regeneration process can reduce energy consumption during regeneration.

## 4. Conclusions

In this study, ZSM-5/PVDF nanofibers for use in cover-free compact PGMEA air filters were synthesized by a method in which core–shell ZSM-5/PVP-PVDF nanofibers were directly electrospun on a mesh substrate followed by the wet etching of PVP in the shell to expose the active sites of ZSM-5. The electrospinning behaviors of the core–shell ZSM-5/PVP-PVDF nanofibers that were electrospun directly on mesh substrates were examined to identify the optimal electrospinning time and RH (20 min and 30–40%, respectively) for fabrication. Based on the wet-etching behaviors of the ZSM-5/PVP-PVDF nanofibers, the optimal etching time for maximum active site exposure was identified as 1.5 h in an EtOH solution (25 vol%). The results of this study imply that nanofibers comprising zeolites with multiple Lewis acid sites should be used in one-time PGMEA air filters that are required to exhibit high adsorption performances, whereas nanofibers containing zeolites with numerous Brønsted acid sites exhibit stable adsorption upon regeneration cycling at a low desorption temperature (80 °C) and should be used in applications involving multiple regeneration cycles. This study indicate that indoor air-quality filters composed of functional nanofibers can be employed for a wide range of potential applications in various fields and should facilitate future research on the design and development of high-performance air-filter materials.

## Data Availability

Data presented in this study are available by requesting from the corresponding author.

## Supplementary Materials

The following supporting information can be downloaded at: https://www.mdpi.com/article/10.3390/nano14131152/s1, Figure S1. FT-IR spectra of the ZSM-5/PVP-PVDF nanofibers before and after etching.
