# Supplementary material for: Electrospinning and Partial Etching Behaviors of Core–Shell Nanofibers Directly Electrospun on Mesh Substrates for Application in a Cover-Free Compact Air Filter
Source: Nanomaterials (Basel). 2024 Jul 5;14(13):1152. doi: 10.3390/nano14131152 (PMC11243679; doi:10.3390/nano14131152)
Supplement: Supplementary file 1 [file nanomaterials-14-01152-s001.zip › nanomaterials-3075265-supplementary.pdf]

Supporting Information

Electrospinning and Partial Etching Behaviors of Core–Shell Nanofibers Directly Electrospun on Mesh Substrates for Application in a Cover-Free Compact Air Filter

Yujung Lee[1], Seungwoo Jung[1] and Ji Sun Yun[1,*]

[1]New Growth Materials Division, Korea Institute of Ceramic Engineering and Technology, 101 Soho-ro, Jinju 52851, Republic of Korea

[*]Corresponding author.

susubin@kicet.re.kr; Tel.: +82-55-792-2675

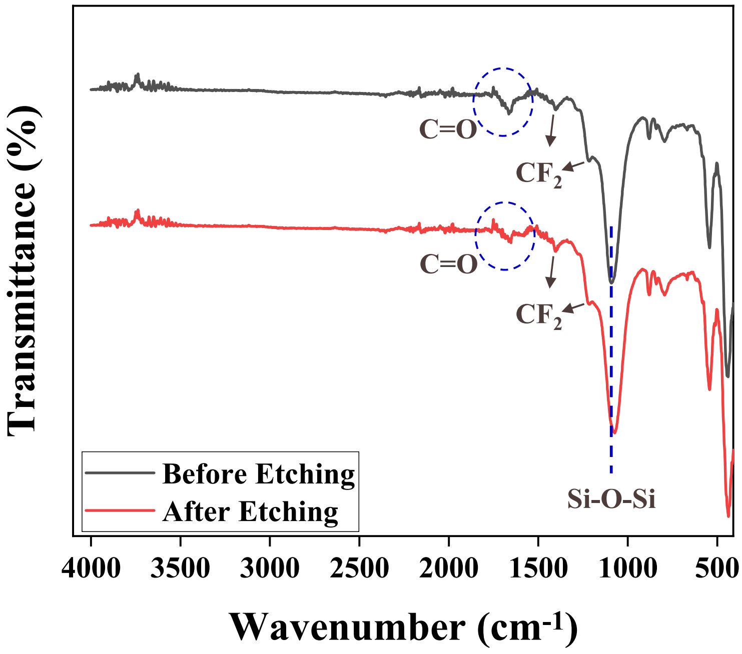

Figure S1. FT-IR spectra of the ZSM-5/PVP-PVDF nanofibers before and after etching.
